# Mr. Bell on the Anatomy, Physiology, and Diseases of the Teeth

**Published:** 1829-10-01

**Authors:** 


THE ANATOMY, PHYSIOLOGY, AND DISEASES OF THE TEETH. By *Thomas Bell*, F.R.S. F.L.S. &c. Lecturer on the Teeth at Guy's Hospital, &c. 1829. Highley.

[ART. I.]

WHEN we look at the BEAU IDEAL which Mr. Lawrence drew of the "ONE AND INDIVISIBLE," a few years ago, and compare it with the actual distracted, divided, and sub-divided state of medicine and surgery, we shall conclude that the talented Painter must have had his organ of IDEALITY strongly excited on that occasion. If physic and surgery be indivisible in themselves, or from each other, how is it that we see them split and splitting in all directions? The Profession appears like a congregation of various wild animals around a carrion. One pecks out the *eyes*; another the *teeth*; a third tugs at the *ears*; a fourth shews its predilection for the *skin*; a fifth (of the true vulture species) gorges on the *liver*; a sixth drains the *gall*-bladder, and, like a generous *church warden*, spirts its nauseous contents on his neighbours; a seventh makes a hearty meal on the *stomach*; an eighth prefers the *bladder*, and, strange to say, converts gravel into gold:—in short, there is scarcely an organ or structure of the human body, which does not furnish a PROFESSORSHIP in physic or surgery—down even to the SPINE, and the more crooked or carious this is, the more to the taste of the professor of spinalogy! The different parts of the body having thus been seized upon by distinct operatives, there is scarcely any thing left for the *pure* physician, the *pure* surgeon—nay, for the GENERAL PRACTITIONER himself! These three classes are fast approaching the seventh age of Shakespeare, and are likely to be soon—

"Sans eyes, sans teeth, sans ears, sans every thing."

Various are the opinions as to the utility or inutility of these divisions and sub-divisions in practice; but it is evident that the Public has already decided the question in their favour. This being the case, we have only to hope that the different Professors will qualify themselves in *general* medical science and literature, before they devote themselves to particular branches of practice. The want of this has, in many instances, brought odium on the professorships; but is now in course of rectification. We apprehend that this "division of labour" in our art has not contributed much to unity of opinion among the artists—nor yet to liberality of sentiment towards each other. In the course of our editorial labours, we have learnt one caution at least—never to set an oculist, aurist, dentist, or other mono-medicaster (we beg pardon for the term) to the task of reviewing his neighbour's work. To do so would be to commit little less than literary homicide! The oculist would be sure to leave his brother oculist without an eye—the dentist would put it out of his neighbour's power hereafter to bite—while the aurist would deafen his collaborateur with censures loud and deep. It is on this account that, uninitiated as we are in the secrets of dental, aural, and ophthalmic surgery, we are forced on the task of reviewing these chef-d'œuvres of art, without the aid of the adepts. Fortunately, we deal but little in criticism, and the operation of analysis is so much a matter of fact process, as to be carried on without the help of Aristotle.

Mr. Bell has long been distinguished as an able practical dentist, and scientific lecturer on dental surgery. Having reputation to *lose* among his professional brethren, it is not likely that he would come before the public, as some authors do, in disguise. Mr. Bell has evidently endeavoured to construct a work of reference for the practitioner, and a text-book for the student, containing "a plain and practical digest of the information at present possessed on the subject—and the results of the author's own investigations and experience." The work being, in great part, an elementary one, is not a proper subject for analysis—particularly the anatomical and physiological portions; but the second part of the volume, containing a description of the diseases of the teeth, the causes of these diseases, and the methods of treatment, will furnish us with ample and useful materials for one, if not two articles in this Journal.

Two opposing doctrines have tended much to produce confusion and misunderstanding in the pathology of the teeth. One sect denied the organization of these bodies, and consequently denied their susceptibility to diseased conditions—the other party maintained the identity of the teeth with the bones, not only in their structure but in their diseases. The principle which Mr. Bell maintains in this work is that, although the structure of the teeth is similar to that of bone, yet the phenomena which the teeth display in disease, are so modified by *the lower grade of their organization*, and the less active condition of their living powers, as, in many cases, to exhibit characters essentially different from those which belong to the analogous diseases of bones. To these obvious causes of discrepancy, may be added, the uniform density of their substance—the absence of cancelli—the peculiarity of their situation, a part being exposed to external causes of disease, protected only by a thin layer of inorganic matter. With these preliminary observations our author proceeds to dental diseases.

### I.—GANGRENE OF THE TEETH, VULGARLY CALLED CARIES.

This is one of the most common dental diseases; but the term is wrong, and calculated to mislead. The disease has not the slightest analogy to true caries of bone. Mr. B. proposes to substitute the term gangrene, a word expressing the real nature of the complaint.

"It usually attacks the crown of the tooth; sometimes, though rarely, the neck; but I believe it scarcely ever makes its first appearance on the root. It invariably shews itself on the external surface of the bone, immediately underneath the enamel, and its existence is, in many cases, first indicated by an opake spot on that substance, occasioned by partial breaking down of its crystalline structure; in others, its presence is shewn by the discoloured bone being seen through the semi-transparency of the enamel. If at this stage of the disease the tooth be sawn through at that part, so as to intersect its centre, a brown mark will be found in the bone, immediately under the opake spot of the enamel, extending more or less into the substance of the tooth, in a line tending directly towards the cavity; it is darkest at the surface, where, from the disease having commenced at that part, its progress is more advanced, becoming gradually lighter towards the centre." 119.

The disease does not begin in the enamel, but the crystalline structure of the latter is deranged by the want of support in the gangrened part beneath. The mortification insidiously increases, and destruction of the bone goes on in a central direction, the part becoming blackish and softened, till at last the enamel itself gives way, and discovers a cavity below. When the internal cavity of the tooth is either actually exposed, or covered only by a softened portion of bone, inflammation spreads to the lining membrane, and produces what Burns emphatically called that "hell o' a' disease"—the TOOTH-ACHE! Thus portion after portion is destroyed, till the whole crown of the tooth is removed, and the roots only remain as dead extraneous matters in the sockets. In this state they often remain stationary for years—but in very irritable constitutions, a cold, a mercurial impregnation, indigestion, or some other cause, will occasion irritation in the socket, followed sometimes by abscess in the alveolus, caries of the bone, or tumours of various kinds—nay, by pains bearing a close resemblance to tic douloureux. In some people, the decay takes place gradually and unaccompanied by pain.

"When the roots are, by the advance of gangrene, rendered mere extraneous bodies in the alveoli, three different actions are set up to effect their removal. In the first place, absorption of the alveoli and gums occurs to such an extent, as gradually to loosen the roots, by depriving them of their support. At the same time also, a deposition of bone takes place at the bottom of the socket, which, by degrees, forces the root into the substance of the gum, until it may often be seen lying horizontally, imbedded in that substance, without any attachment to the bone, or the slightest lodgment in the socket; and in the third place, the roots themselves undergo absorption at their extremities, so that, at length, a very small portion only is, in many cases, found to remain." 122.

*Causes of Dental Gangrene.* Hunter and Blake approached very near to the true etiology, but still, according to Mr. B. they fell short of the mark. The following is Mr. Bell's doctrine in a very few words.

"When, from cold or from any other cause, a tooth becomes inflamed, the part which suffers the most severely is unable, from its possessing comparatively but a small degree of vital power, to recover from the effects of inflammation; and mortification of that part is the consequence." 124.

That the bony structure of the tooth is *liable* to inflammation Mr. B. considers to be proved, not only by the identity of symptoms with those of osseous inflammation elsewhere, but by the fact, that distinct patches are often found in teeth, injected with the red particles of blood. The continued and invariable progress of dental gangrene is to be accounted for by the structure of the organ and the seat of the disease. When a portion of tooth is killed by inflammation, it excites an increased action in the vessels of the surrounding portion of bone; but the ratio of vitality here, being very low, the diseased part cannot be thrown off, as in other bones—hence the continued extension of the dental gangrene. The common supposition, that this gangrene may arise from merely external causes acting on the enamel is, Mr. B. observes, fallacious.

*Predisposing and Remote Causes.* Hereditary predisposition is the most common and the most remarkable among the *first* class. It often happens that this tendency exists either in the whole or great part of a family of children, where one of the parents had been similarly affected. Our author has often seen the very same tooth, and the same part of the tooth, affected in several individuals of the same family and about the same age. The whole list of infantile diseases, which occur during the formation of permanent teeth, are so many predisposing causes of dental gangrene—and not the diseases alone, but some of the remedies employed in their cure—as mercury, for example, injudiciously used. The strumous constitution is very liable to this disease.

Amongst the *remote* causes, or those which produce a deleterious change in the constitution of the teeth, subsequent to their formation, one of the most extensive, Mr. B. thinks, is mercury. But fevers of every kind, dyspepsies—in short, all constitutional disorders of any protracted continuance, must be classed amongst the remote causes of dental gangrene.

*Exciting causes.* Whatever tends to induce inflammation in the teeth may become an exciting cause. The most frequent are sudden vicissitudes of temperature, whether atmospheric, or from taking very hot or very cold substances into the mouth.

"As a general rule it may be observed, that whatever is placed in contact with the teeth, either so much higher or lower than the natural temperature of the body, as to produce pain, may probably prove to be the exciting cause of the disease; thus, drinking very hot fluids on the one hand, and, on the other, taking ice, without the precaution of preventing it from lying in contact with the teeth, are, I am convinced, fertile sources of disease in these organs. When the extremely dense, solid structure of the teeth is considered, it will not appear wonderful that this result should occur from change of temperature in a part, the unyielding nature of which precludes the possibility of expansion and contraction, not only in the vessels of its substance, but also in the membrane filling its cavity." 131.

The exposure of the bony structure of a tooth by the destruction of its enamel, whether the consequence of imperfect formation, accidental fracture, or the lateral pressure of the teeth against each other, is another exciting cause of dental gangrene by inflammation. The general notion, that one decaying tooth will communicate the disease to another, is, Mr. B. avers, erroneous. Mr. B. in the 10th vol. of the Med.-Chir. Transactions, has endeavoured to prove that "this is totally inconsistent with fact, with the chemical composition of the teeth, and with the cause of gangrene, as already explained." There can be no chemical agent, he observes, evolved in the destruction of one tooth, which can possibly occasion the decomposition of another. "Nor, as the bone of a perfect tooth is completely protected by the enamel, could any substance of an irritating nature (supposing such to be evolved) come in contact with the living portion of the sound tooth." This is a serious point of discrepancy among the "PROFESSORS OF DENTISTRY," and we wish they would settle it without sacrificing the grinders of the community at large. If Mr. Bell's doctrine be true, we are every day losing our teeth unnecessarily by extraction—if he be wrong, we shall lose them by the spread of the disease! About the following piece of advice there can be little difference of opinion.

"Whatever tends to irritate and inflame the gum, must in a greater or less degree produce a corresponding irritation in the teeth, from the close connexion which subsists between them; and hence the accumulation of tartar, portions of food remaining between the teeth, or any similar circumstance, may possibly become an exciting cause of gangrene; and this, not only by means of inflammation propagated through the gum, but also by the exposure of the necks of the teeth to external agents, in consequence of the absorption of the gum and alveolar processes. The necessity of keeping the teeth in a constant state of cleanliness, and freedom from all extraneous substances, will be particularly insisted upon in another place, but deserves to be alluded to here, as a means of preventing the occurrence of gangrene from the causes just-mentioned." 135.

*Precautions against Dental Gangrene.* Under this head our author has laid down many rules, which are, no doubt, judicious. In the first place, fluids should never be taken into the mouth either so hot or so cold as to produce pain. In the second place, when any inflammation is set up in a tooth, it should be immediately reduced by the application of leeches to the gum, as near as possible to the part in pain. This should be repeated, and the bleeding encouraged by holding warm water in the mouth.

"When, from the want of room in the maxillary arch, the teeth are so crowded as to press with considerable force against each other, this pressure should be removed, by passing a very thin file between those which are, in the greatest degree, subjected to it. These will generally be the incisores. As a very small portion only will be required to be removed, the file should be as thin as possible, and exceedingly fine. It is of the utmost consequence that the whole thickness of the enamel should *not* be taken away from either of the teeth between which the file is passed, but that a perfect covering of this substance should still be preserved, to protect the bone from external agents; and it is never to be forgotten that this operation is only to be had recourse to, as the least of two evils, and therefore requires care and judgment in deciding upon the propriety of its adoption. In the mode of its application also, care must be taken that the force used shall be so regular and moderate, as not to risk the chipping off or fracturing of the enamel. Should a bicuspid, or even a molar tooth be already considerably diseased, it will, in many cases, be sufficient to remove it; for, by degrees, the other teeth will tend towards the vacancy, so as to take off their mutual pressure upon each other, and thus obviate the necessity of filing." 137.

*Treatment of Dental Gangrene.* When Gangrene has actually occurred in a tooth, it is very probable that the progress is not to be stopped by art. In the earliest stage, where the only indication of the disease is an opake or darkish spot on the enamel, and before the subjacent bone has become softened, a careful removal of the diseased spot should, if possible, be effected. By this procedure, the exciting cause of irritation is withdrawn. The excision should be confined to the dead portion. The file is a dangerous instrument, as it cannot be restricted to the decayed part, but must necessarily remove a portion of sound tooth. Yet it is the only instrument which, at an early period of the disease, and where the enamel is not weakened by softening down of the bone beneath, can effect the purpose. It must, therefore, be worked with great caution. If the bone be still very hard, it should be taken away by strong steel brooches, having three sided points, and being of various sizes.

*Filling the Cavity produced by Gangrene.* This operation is often necessary, to exclude the external causes of the original disease—and, when carefully performed, it is, in many cases, perfectly effectual in arresting the further progress of the malady.

"The substance best adapted for this purpose is pure gold, both on account of its ductility and the toughness of its texture, and, more particularly, because it is not liable to become oxydized. Tin and silver are frequently used, but they combine too readily with oxygen to be sufficiently durable. Lead is still more objectionable, as its salts are readily soluble in the saliva; and where many teeth have been filled with it, particularly if the decay is extensive, and there is consequently a considerable surface of the metal exposed to the action of that solvent, the stomach may probably become materially disordered by it. Platina is free from these objections, but, in addition to its being usually alloyed to render it malleable, it is less tough and ductile than gold. Metallic compounds, fusible at a very slight degree of heat, have been recommended, and are now used, not only on the continent, but in some instances in this country. These are decidedly objectionable, not only for the reasons already given for rejecting other oxydizable metals, but also from the very imperfect manner in which the metal runs into the inequalities of the cavity, and the inadequate protection which it consequently affords.

"The gold should be beaten very thin for small cavities, and rather thicker for larger ones, but in no case should it be so thick as not to be easily pressed into any form, and forced into all the irregularities of the cavity. It should also be thoroughly annealed after it is beaten to the proper thickness, in order to deprive it of the elasticity which it had received from the hammer.

"A sufficient quantity of gold is to be taken in one piece to complete the stopping of each tooth; for if too small a piece be used at first, and the deficiency be supplied by a second, the latter will be liable to be removed by the food in mastication. The gold is to be forced by degrees into the tooth, taking care that the first portion is placed in contact with the bottom of the cavity, and that every succeeding portion is firmly pressed against that which preceded it, so that every irregularity of the excavation shall be filled. If this be not attended to, and the whole of the gold be carelessly and slightly pressed into the cavity, and the degree of force necessary to its complete consolidation be afterwards given to the whole mass at once, it will often happen that the gold forms a hard solid body at the orifice, and a short space within it; whilst the bottom of the cavity is left wholly unprotected. The gold is therefore to be gradually and firmly introduced into the cavity, every portion receiving the degree of force necessary to its consolidation, until the tooth is completely filled, and the gold forms a hard, unyielding, and continuous mass. Any superfluous metal is then to be cut off, and the surface polished with a little steel burnisher." 146.

We shall not enter into a description of the various instruments necessary for this operation. Mr. Bell has given Mr. Koecker a sly rub about the number which the latter employs—but this we shall pass. Mr. B. remarks that it is not uncommon for dentists to fill teeth, where the bone has become softened to such an extent as to occasion pain on being pressed, and where the total removal of the decay would expose the membrane of the tooth. In these cases, the dead bone is forced into contact with the membrane, or the gold is pressed upon the naked nerve. In both cases, the effect is the same. Inflammation and great suffering are generally the result. These sufferings may sometimes be obviated by removing the stopping, and introducing a solution of the nitrate of silver by means of a camel's hair pencil, the cavity having been previously cleaned and dried. If the inflammation has continued till the periosteum is thickened and matter has begun to form, the tooth must be extracted. As a general rule, the hollow of a tooth should never be filled at a time when pressure of the gold occasions that peculiar pain arising from contact with the membrane.

The actual cautery, sulphuric acid, and other corrosive caustics, have been employed to destroy the nerve of the tooth; but they are all objected to by Mr. Bell. The best application, for the purpose of obtunding the sensibility of the membrane or promoting its absorption, is alkohol, or solution of nitrate of silver. Camphorated alkohol is preferred by our author. The sensibility obtunded the process of stopping may be resorted to.

TOOTH-ACHE. This is one of the most excruciating pains to which humanity is liable. The sensation is *sui generis*—scarcely is tic douloureux more agonizing. The sympathetic affections to which it gives rise are very numerous. The pain often *appears* to be seated in parts remote, as the face, scalp, ear, jaw, neck, shoulder, and even along the whole arm. That of the ear, however, is the most common of the sympathetic pains, from exposure of the nerve supplying the inferior dens sapientiæ. Cases of idiopathic ear-ache are comparatively rare. There are few applications of much use in this distressing malady. "The following are perhaps the most useful."

"℞. Aluminis, ʒi.

Spir. Æther. Nitrici f ℥ss. Misce.

"℞. Acid. Muriat. f ʒss.

Aquæ distillatæ f ʒij. Misce.

"℞. Argenti Nitrat. gr. i.

Aquæ distillatæ f ʒi. Misce.

"A small bit of lint, wetted with either of these liquids, may be frequently introduced into the cavity, which should be carefully dried previous to each application.

"It is however only by treating this affection, as nearly as the circumstances will admit, upon the same principles as inflammation in other parts, that any relief can, in general, be rationally expected. In those attacks therefore in which the inflammation is considerable, and there is any particular reason for preserving the tooth, leeches should be freely and repeatedly applied to the gum, the bleeding being encouraged by repeatedly holding warm water in the mouth. After the inflammation and pain are thus reduced, should the nerve be only in a small part exposed, the means already mentioned for diminishing its sensibility may be had recourse to. But the hope of relief which these remedies may, from occasional success, hold out, is in most instances completely fallacious, and the extraction of the tooth can alone be depended upon." 157.

The operation of excising the crown of the tooth, as practised by Mr. Fay, is reprobated by our author in the strongest terms, as attended, to his knowledge, with injurious, nay, even fatal consequences. "It is an operation irrational in its principle, often useless in its immediate effects, and in its consequences most pernicious."

"It has always appeared to me to place the operator in a dilemma of evils. The object, I presume, is to cut through, or, more properly, to break off the tooth so low as to remove the whole of the crown, including the cavity which contains the pulp or membrane. If this object be effected, the consequence is that the dead roots remain in the alveoli; and these, if not immediately productive of pain, may yet be expected to occasion much future suffering as extraneous irritating bodies. Every one knows what is the usual result of the existence of dead roots in the jaw, when they have been left either by accident in an attempt at extraction, or by the gradual decay of the crown; and it is surely too much to adopt as an useful operation, that which every one deprecates as an accidental occurrence.

"If, on the other hand,—as indeed it frequently happens,—the object aimed at be not fulfilled, the case is placed in a situation incomparably worse than before, the nerve being still more exposed, and the hope of the ready and easy extraction taken away by the loss of that part of the tooth which would have afforded a solid support for the instrument." 159.

*Total Necrosis of the Teeth.* The causes of this state are various. A blow sufficiently forcible to break the vessels entering the foramen of the root—inflammation occasioning separation of the internal membrane—fever—the immoderate use of mercury—and many other causes, may produce total dental necrosis. The incisores or cuspidati are the teeth most subject to the disease. When the tooth has been sometime dead, it begins to assume a darkish colour, which gradually increases to black. Mean time inflammation takes place in the socket—the gum becomes spongy—and there is a constant discharge of matter through one or more openings opposite the root. Next come on absorption of the alveolar process, and loosening of the tooth.

"In the earlier stages of necrosis, free and repeated scarification of the gum, followed by the application of a strong astringent lotion, will be found very beneficial; I have also employed the injection of a solution of sulphate of zinc underneath the gum into the enlarged alveolus, by means of the extremely fine tube of a syringe, with much advantage in checking the formation of pus, and giving tone to the gums. It is however obvious that when an extraneous body, like this, exists in the socket, nothing short of its removal can effect a cure." 167.

Attempts to cure tooth-ache by extracting the tooth and then replacing it, bring on a state precisely similar to the above. Mr. Fox, who first proposed this procedure, candidly acknowledged its entire failure. The transplanting of teeth is deprecated by Mr. Bell, and is, we should think, now entirely given up, after the dreadful consequences which are known to have resulted from it.

*Exostosis of the Teeth.* A deposit of bone occasionally takes place on the root of a tooth precisely similar to exostosis in other bones. The new substance is of a particularly hard dense texture, of a yellowish transparent hue. It is occasioned by an increased and irregular action of the vessels—a kind of chronic inflammation, the result, in most instances, of incipient gangrene.

"Hence its progress is so tardy, that, in most instances, the enlargement of the alveolus by absorption, almost keeps pace with the deposition of new bone; and the pressure which the latter produces is so trifling and gradual, as to occasion no more than a slight, though continued uneasiness; and it is only when the caries has extended to the cavity, and tooth-ache is produced by the exposure of the membrane, that the patient is induced to lose the tooth, and that the true cause of the previous affection is ascertained. In other instances the continued irritation occasions thickening of the periosteum, and afterwards suppuration, and the case becomes one of simple alveolar abscess." 174.

The pressure arising from this enlargement of the root sometimes produces an affection resembling tic doulouréux. The following case is important, as it shews how small an exostosis will produce the most painful symptoms.

"Mr. ——— had for some months suffered severe and frequent paroxysms of pain on the left side of the face, apparently commencing in the second inferior bicuspis, and darting through the lower jaw to the ear, and upwards to the temple. The pain resembled tic douloureux in the nature of its attacks, but was evidently produced by a local rather than a constitutional cause, from the paroxysms occurring without the least periodical regularity, and from their being excited by the application of heat to the teeth of that part. On the most careful examination, however, I could not discover the least appearance of caries in any of the teeth, and I therefore ordered leeches to be applied to the gum, and directed aperient medicines and abstinence from all stimulating food. This plan was productive only of temporary and partial relief, and in about two days the pain was as severe as ever. Finding that a smart blow on the second bicuspis produced a more painful sensation than on any other tooth, I determined on extracting it, and found the extremity enlarged by a deposition of bone, giving to it a slightly bulbous shape, but not larger than the tip of a small quill. The newly added bone was yellower and more transparent than the original structure, as is generally the case in this disease. The removal of the tooth was followed by immediate and entire relief." 176.

Here we shall close our first article. In our next paper we shall proceed with various other diseases of the teeth. This short analysis will demonstrate to the general practitioner, as well as to the professed dentist, the importance and the value of Mr. Bell's book, which we can conscientiously recommend to all classes of our readers.

